# The Parish of St. Pancras and Its Lunatic Poor

**Published:** 1856-04-01

**Authors:** 


### THE PARISH OF ST. PANCRAS AND ITS LUNATIC POOR.

At a general meeting of the directors and guardians of the poor of the parish of St. Pancras on Tuesday, an official letter from the Secretary of the Commissioners of Lunacy was read to the board. It was accompanied by a Report by Mr. Gaskell, one of the Commissioners in Lunacy, upon his recent inspection of the wards appropriated to the insane and idiotic inmates of St. Pancras Workhouse, and the directors were urged to attend to its recommendations without delay. Mr. Gaskell's Report disclosed a lamentable state of disorder and neglect as prevailing in the wards inspected. He speaks of them as much overcrowded and dirty. In certain wards which were in a former report described as inconveniently crowded when there were 13 men and 16 women in them, there are now 21 men and 45 woman. Eight patients are unprovided with beds; two men slept on beds placed on the forms, and two on the floor of the padded room. The report expresses regret that previous suggestions of the commissioners have been utterly disregarded. The Report, after detailing numerous evils existing in the insane wards of St. Pancras Workhouse, concludes by drawing attention to the following, which they recommend:—1. That no recent case of insanity be detained in the workhouse. 2. That chronic cases on whom restraint or seclusion are imposed be removed to an asylum. 3. That the number of inmates in the idiotic wards be forthwith diminished. 4. That more means of exercise and occupation be provided, and that a larger number of patients be allowed to attend divine service. 5. That a bath be supplied to the women patients. 6. That the better means of washing be provided for the men, and a larger supply of towels and sheets for both men and women. 7. That suitable beds to prevent bed sores be purchased. 8. That better means of cleansing soiled bedding be adopted, and a larger number of bed-ticks be supplied. 9. That the wire-work be removed from the windows in the men's ward. 10. That the women be supplied with suitable books. After the presentation of the Report, it was resolved to appoint a committee of five members, to inquire into the grounds of its allegations. A letter was also read from the Poor Law Board, urging the directors of the poor to lose no time in giving effect to the measures necessary to remedy the serious evils shown in Dr. Bence Jones's Report to exist at St. Pancras Workhouse.—*Times*, March 6th, 1856.

